# Advances in non‐germ cell tumours of the testis: focus on new molecular developments in sex cord‐stromal tumours

**DOI:** 10.1111/his.70006

**Published:** 2025-12-12

**Authors:** João Lobo, Andres M Acosta

**Affiliations:** ^1^ Department of Pathology Portuguese Oncology Institute of Porto (IPO Porto)/Porto Comprehensive Cancer Center Raquel Seruca (P.CCC), R. Dr. António Bernardino de Almeida Porto Portugal; ^2^ Cancer Biology and Epigenetics Group, IPO Porto Research Center (GEBC CI‐IPOP), Portuguese Oncology Institute of Porto (IPO Porto)/Porto Comprehensive Cancer Center Raquel Seruca (P.CCC), R. Dr. António Bernardino de Almeida Porto Portugal; ^3^ Department of Pathology and Molecular Immunology, ICBAS ‐ School of Medicine and Biomedical Sciences University of Porto Porto Portugal; ^4^ Department of Pathology Indiana University Indianapolis Indiana USA

**Keywords:** granulosa cell tumour, Leydig cell tumour, Sertoli cell tumour, sex cord stromal tumours, testicular cancer

## Abstract

Testicular sex cord‐stromal tumours (TSCSTs) represent ~4%–8% of all testicular neoplasms. Most show a Leydig or Sertoli cell phenotype and exhibit benign clinical behaviour. However, a subset of ~10% is malignant and clinically problematic, as TGCTs do not respond to systemic therapy. Classification of TSCSTs has relied on morphology, with several entities being defined based on their resemblance to more common ovarian counterparts (e.g. granulosa cell tumours). In recent years, multiple clinicopathologic and molecular studies have improved our understanding of the mechanisms that underlie pathogenesis and progression in TSCSTs, providing data that can be useful to refine classification and prognostication. In this review, we summarise the major recent advances in TSCSTs, focusing on molecular alterations and biomarkers relevant for diagnosis, classification and prognosis.

## Graphical abstract

In this review, we summarise the major recent advances in Testicular Sex Cord‐Stromal Tumours, focusing on molecular alterations and biomarkers relevant for diagnosis, classification and prognosis.

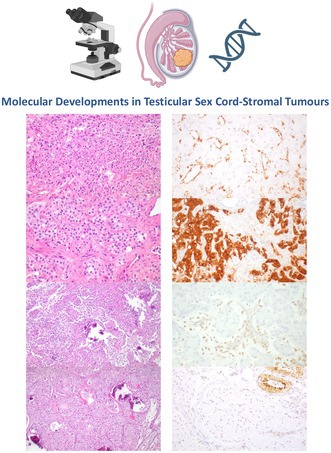

**Key points**

- Classification of TSCSTs has mostly relied on morphology, but recent contributions have contributed to an improved understanding of these tumours.
- Several molecular biomarkers have been introduced that refine the diagnosis and categorization of TSCSTs.
- Additionally, several molecular alterations have been described that improve prognostication of TSCSTs, which is much needed because their clinicopathologic features are suboptimal predictors of clinical behaviour.

**Abbreviations**
ACTH: adrenocorticotropic hormone
FAP: familial adenomatous polyposis
GUPS: Genitourinary Pathology Society
ISUP: International Society of Urological Pathology
LCCSCT: large cell calcifying Sertoli cell tumour
LCT: Leydig cell tumour
MGST: myoid gonadal stromal tumour
NOS: not otherwise specified
PARP: poly(ADP‐ribose) polymerase
TGCT: testicular germ cell tumour
TSCST: testicular sex cord‐stromal tumour

## Introduction

The testis proper is composed of several cell types (germ cells, Sertoli cells, Leydig cells, peritubular myoid cells, among others), all of which can give rise to neoplasms.^1^ Germ cell tumours, the most common testicular neoplasms in all age groups, derive from germ cell precursors that originate in the extraembryonic mesoderm of the yolk sac.^2^ A smaller subset of testicular tumours arises from (or differentiates to) components of the testicular stroma or sex cord derivatives, which originate from the embryonic mesoderm and coelomic epithelium, respectively.^3^

The low incidence of testicular neoplasms, which account for ~1% of newly diagnosed male cancers worldwide,^4^ creates challenges for proper classification because exposure to them is limited in most pathology practices.^5^, ^6^ Given that more than 90% of testicular neoplasms are of germ cell origin (i.e., testicular germ cell tumours—TGCTs),^1^ these challenges are even greater for testicular sex cord‐stromal tumours (TSCSTs). These comprise 4%–8% of all testicular neoplasms,^1^, ^7^, ^8^ with most showing Leydig or Sertoli cell phenotype.^7^, ^9^ From a clinical perspective, ~90%–95% of TSCSTs overall are benign and therefore cured by complete surgical excision, with testis‐sparing surgery being feasible in selected patients.^9^ However, the remaining 5%–10% of TSCSTs that exhibit malignant behaviour represent an important challenge for patient management given their consistent unresponsiveness to chemo‐ and radiotherapy.^10^, ^11^ Another major issue with these tumours is that their clinicopathologic features are suboptimal predictors of malignant potential. Recent advances in our understanding of the molecular and biologic features of TSCSTs may help to improve the identification of those with malignant potential and uncover therapeutic targets.^12^ Herein, we summarise the current knowledge on TSCSTs, focusing on new molecular findings and their potential impact on nosology and clinical management. A brief compilation of the most important clinicopathologic features of TSCSTs is provided in Table 1.

**Table 1:** Clinicopathological features of testicular sex cord–stromal tumours

Tumour	Clinical behaviour	Syndromic associations	Cytomorphology	Stroma	Intratubular component	Calcifications	Inflammatory infiltrates	Nuclear beta‐catenin	Molecular alterations
Leydig cell tumour	~90% benign; ~10% malignant	*HLRCC* (rare), Klinefelter syndrome	Large eosinophilic epithelioid/polygonal cells	Minimal, typically imperceptible. In some cases more abundant, hyalinised or oedematous	No	Rare, typically small and scattered	No (very rare)	Yes (in a subset; typically focal or multifocal)	*LHCGR* mutations (largely in paediatric cases), *FH* variants (enriched in aggressive cases), *MDM2* amplification (enriched in aggressive cases), *CTNNB1*, multiple chromosomal aneuploidies (enriched in aggressive cases)
Testicular tumours of the adrenogenital syndrome	Benign	Congenital adrenal hyperplasia	Large eosinophilic epithelioid/polygonal cells	Moderate, fibrous	No	No	No	No	NA
Sertoli cell tumour, not otherwise specified	~90% benign; ~10% malignant	FAP (rare)	Intermediate‐sized epithelioid cells with eosinophilic cytoplasm; frequent univacuolated (signet ring‐like) cells	Moderate to abundant; collagenous with variably hyalinisation	No	No	No	Yes (>90% of primary tumours, diffuse)	*CTNNB1* mutations (very common), *APC* mutations (rare)
Inflammatory and nested testicular sex cord tumour	Malignant	No	Intermediate‐sized epithelioid cells with eosinophilic granular to clear cytoplasm	Moderate to abundant; thick collagenous	No	No	Yes (mixed, containing lymphocytes, plasma cells, eosinophils and neutrophils)	No	*EWSR1* rearrangement; *EWSR1::ATF1*
Large cell calcifying Sertoli cell tumour	~90% benign; ~10% malignant	Carney complex (~10–40%), PJS (debated), NF1 (rare)	Large eosinophilic epithelioid/polygonal cells	Typically myxoid	Yes (in a subset)	Yes (mulberry‐like, with concentric laminations)	Yes: neutrophilic (within the stroma), lymphocytic (typically peripheral)	No	*PRKAR1A* variants (both in sporadic and syndromic tumours)
Large cell hyalinising Sertoli cell neoplasia	Benign	PJS (invariable)	Large eosinophilic epithelioid/polygonal cells	Collagenous and often hyalinised (between the tubules)	Yes (pure intratubular with rare exceptions)	Yes (common; not mulberry‐like)	No	No	*STK11* alterations (inferred by loss of LKB1 expression)
Adult granulosa cell tumour	~90% benign; ~10% malignant	No	Variable, from small to intermediate‐sized epithelioid or round cells to spindle cells	Variable, typically fibrous	No	No	No	No	*FOXL2* p.C134W in rare cases
Juvenile granulosa cell tumour	Benign	No (some associated with gonadal dysgenesis)	Small round cells with scant eosinophilic to clear cytoplasm	Fibrous, consisting or prominent bands	Uncertain (small nests may represent tumour cells confined to prepubertal seminiferous tubules)	No	No	No	Loss of chromosome 10 (~60%)
Spindle cell gonadal stromal tumours (including fibroma/thecoma and MGST)	Benign	No	Bland spindle cells	Variably prominent, collagenous	No	No	No	No	Multiple chromosomal gains
Mixed TSCST	Mostly benign	No	Combination of sex cord elements with immature Sertoli cells and bland spindle cells; combination of Sertoli cells and Leydig cells (very rare)	Typically scant	No	No	No	Yes (in a small subset; restricted to Sertoli/sex cord cells)	Multiple chromosomal gains; *CTNNB1* mutations in a subset

FAP, familial adenomatous polyposis coli; HLRCC, hereditary leiomyomatosis and renal cell carcinoma; MGST, myoid gonadal stromal tumour; NA, Not applicable; NF1, neurofibromatosis type 1; PJS, Peutz‐Jeghers syndrome; TSCST, testicular sex cord–stromal tumour.

In this review, except for relatively specific or clinically relevant markers, little space will be dedicated to discussing immunohistochemistry. This is because the ‘classic’ TSCST markers such as SF1 and inhibin, among others, are largely non‐specific and consequently not very helpful for distinguishing between TSCST types. The embryonic sex cords derive from the coelomic epithelium^13^; therefore, as a general concept, tumours with a sex cord phenotype (e.g., Sertoli cell tumours) typically express some of the markers that are also positive in mesothelial cells (e.g., WT1) as well as transcription factors associated with differentiation of primitive sex cord cells to Sertoli and granulosa cells (e.g., SOX9 and FOXL2, respectively).^14^, ^15^ In contrast, Leydig cells and other components of the stromal compartment are thought to derive largely from the mesoderm; hence, stromal tumors are often negative for ‘sex cord markers’ such as WT1, SOX9 and FOXL2. Notwithstanding these general concepts, there are multiple exceptions, and these markers should not be considered entirely specific for any TSCST type. For a comprehensive assessment of ‘classical’ TSCST markers the readers are referred to the study performed by Lau *et al*.^14^

## Leydig Cell Tumour (LCT)

LCT represents approximately 70% of all TSCSTs,^7^ comprising 3% of adult and 4%–9% of paediatric testicular tumours.^16^ Its incidence is biphasic, with a peak in early childhood (~20% of cases) and another in middle adulthood (~80%). Patients may present with symptoms of excess steroid sex hormone production (androgens and/or oestrogen), such as gynecomastia and isosexual precocious pseudopuberty in children.^17^ In contrast, in adult patients, most LCTs present as a testicular mass that is sometimes associated with local symptoms such as scrotal pain.^16^

**Figure 1:**
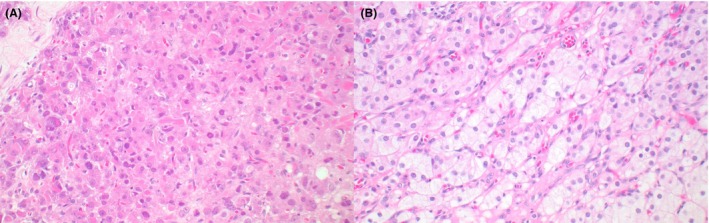
Leydig cell tumour. (**A**) Leydig cell tumour with prominent Reinke crystals and intracytoplasmic hyaline globules. (**B**) Leydig cell tumour with ‘lipidised’ cells containing lipid‐laden microvesicles

Typical LCTs are solid, composed of sheets and nests of polygonal cells with an abundant amount of bright eosinophilic granular cytoplasm and round regular nuclei with a single visible nucleolus. Nuclei with ‘ground‐glass’ appearance and scattered cells with intracytoplasmic eosinophilic globular inclusions are often identified. Rod‐shaped intracytoplasmic Reinke crystals represent a pathognomonic finding (Figure 1A). Lipofuscin pigment granules are often present in scattered cells, possibly as a consequence of cholesterol metabolism. The stroma is characteristically scant and contains delicate branching capillaries. However, multiple morphologic variations have been described, including tumours with ‘lipidised’ cells (Figure 1B), more abundant stroma, scattered calcifications and microcystic areas.^18^, ^19^ The immunoprofile of LCTs is characterised by a high frequency (>95%) of expression of SF1 and inhibin, whereas calretinin is less sensitive. Markers expressed in sex cord cells, such as WT1 and FOXL2, are mostly negative.^14^

**Figure 2:**
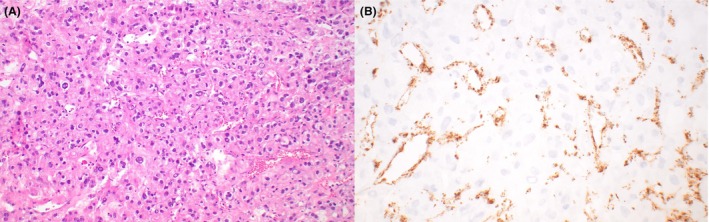
Fumarate hydratase‐deficient Leydig cell tumour. (**A, B**) Leydig cell tumour with marked atypia (**A**) and loss of expression of fumarate hydratase (**B**)

**Figure 3:**
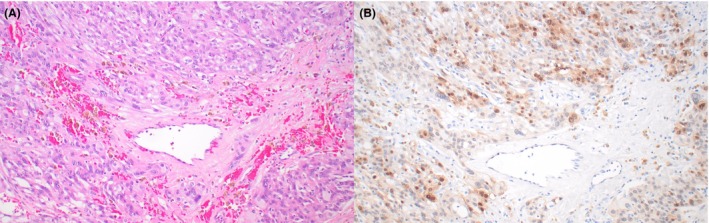
Leydig cell tumour with *CTNNB1* alterations. (**A, B**) A subset of Leydig cell tumours (**A**) harbour *CTNNB1* variants (largely involving exon 3). In these tumours, the *CTNNB1* alterations are typically subclonal, which correlates with focal or multifocal nuclear expression of beta‐catenin (**B**)

Over the last two decades, a number of studies have suggested that LCTs of prepubertal children and adult patients show some significant molecular differences. Specifically, LCTs in children presenting with precocious isosexual pseudopuberty typically harbour hotspot gain‐of‐function mutations in codon 578 of the gene that codes for the LH and choriogonadotropin receptor (*LHGCR*).^20^, ^21^ This hotspot *LHCGR* variant (p.D578H) results in constitutive activation of MAPK pathway signalling and internalisation of the LH receptor.^21^ Although variants with similar functional consequences have occasionally been described in LCTs of adult patients, including a tumour with a hotspot codon 201 *GNAS* mutation, most tumours in this age group do not harbour alterations associated with MAPK pathway activation.^22^, ^23^, ^24^ In adult patients, loss‐of‐function mutations in fumarate hydratase (*FH*), including some of germline origin, and gain‐of‐function *CTNNB1* variants, have been described instead.^23^, ^24^ *FH* alterations seem to be enriched in tumours with aggressive histopathologic features or aggressive clinical behaviour, suggesting that loss of FH expression could be a useful prognostic marker in primary tumours with adverse histopathologic findings (Figure 2A,B).^23^ The *CTNNB1* alterations identified in LCTs appear to be largely subclonal and result in focal or multifocal expression of nuclear beta‐catenin (Figure 3A,B), an important difference with Sertoli cell tumour not otherwise specified (NOS; see below).^25^ Amplifications of MDM2 and CDK4 are recurrent in clinically malignant cases, suggesting that they represent events associated with progression. ^26^, ^27^

Approximately 10% of LCTs are clinically malignant and metastasize to retroperitoneal lymph nodes, bone or viscera.^10^ Risk stratification of LCTs has been an area of interest since patients with primary LCTs deemed aggressive based on histopathologic findings could be considered for upfront retroperitoneal lymph node dissection or more intensive surveillance.^9^, ^11^, ^28^ However, prediction of malignant behaviour in LCTs is challenging and relies on the assessment of multiple clinical and histopathological parameters. Although there is no universally accepted and clinically validated tool for risk assessment, most systems proposed to date include at least some of the following parameters: lymphovascular invasion, necrosis, tumour size, infiltrative pattern, extratesticular extension, cytologic atypia, mitotic activity, proliferation index (Ki67) and patient age.^17^, ^26^, ^29^, ^30^, ^31^ These systems have important limitations because there are cases without aggressive histopathologic features that demonstrate malignant clinical behaviour, and the evaluation of most parameters is subject to interpretation (e.g., lymphovascular invasion and invasive growth). Also, the predictive value of the individual parameters is not weighted, even though some findings (e.g., tumour necrosis) are probably more worrisome than others (e.g., cytologic atypia). Therefore, there has been significant interest in identifying molecular biomarkers predictive of malignancy. Studies have described enrichment for *FH* deficiency, *MDM2* amplification and widespread aneuploidy in clinically malignant and/or histologically aggressive LCTs.^23^, ^26^, ^27^ Based on these findings, assessment of primary LCTs with aggressive histologic features for *FH* deficiency and *MDM2* alterations has been recommended. Additionally, interrogation of FH status in metastatic LCT is desirable.^32^ *TERT* fusions have also been demonstrated in three of seven metastatic LCTs, suggesting that they may represent a predictive biomarker.^33^

## Testicular Tumour of the Adrenogenital Syndrome

**Figure 4:**
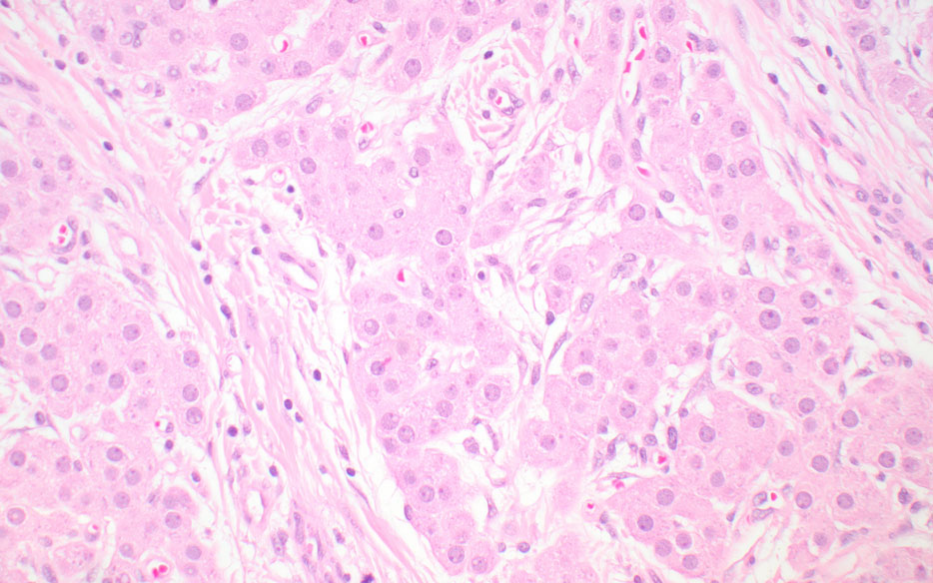
Testicular tumour of the adrenogenital syndrome. These neoplasms occur in patients with congenital adrenal hyperplasia as multifocal, bilateral lesions typically centred in the hilum. They show significant morphologic overlap with Leydig cell tumour; however, some subtle differences have been described. For instance, these tumours lack Reinke crystals and often contain delicate intracytoplasmic basophilic granules (seen in the picture)

Tumours morphologically similar to LCTs that occur in patients with congenital adrenal hyperplasia have been termed testicular tumours of the adrenogenital syndrome and testicular adrenal rest tumours.^34^, ^35^, ^36^ These may be the first manifestation of the syndrome in ~18% of patients^37^ and often consist of multifocal and bilateral tumours centred in the testicular hilum.^37^, ^38^, ^39^, ^40^ They are composed of solid nests and sheets of polygonal cells with abundant eosinophilic cytoplasm embedded in a collagenised stroma.^38^ Tumour cells contain lipofuscin pigment and, sometimes, intracytoplasmic basophilic granules (Figure 4).^37^ The presence of Reinke crystals has not been documented.^37^ Also, unlike LCTs, these tumours have been reported to lack expression of androgen receptor; instead, they are commonly positive for CD56 and synaptophysin.^41^ These tumours are thought to be derived from steroid cells located in the testicular hilum (similar to steroid cell tumours in the ovary), whose excessive growth is driven by the elevated levels of adrenocorticotropic hormone (ACTH) present in patients with congenital adrenal hyperplasia. Of note, testicular tumors of the androgenital syndrome often show a variable degree of regression after initiation of glucocorticoid replacement therapy.^42^, ^43^ The latter phenomenon suggests that they may not always represent a neoplastic process.

## Sertoli Cell Tumour, Not Otherwise Specified (NOS)

Sertoli cell tumour NOS is the second most frequent type of TSCST, after LCT.^7^ The median patient age at presentation is ~39 years, but they occur in a population that ranges from prepubertal children to elderly men. Approximately 90% behave indolently, and the remaining 10% are clinically malignant^44^; however, these figures predate the description of a candidate new entity driven by gene *EWSR1* fusions that has been frequently classified as malignant Sertoli cell tumour, NOS in the past (discussed below).

**Figure 5:**
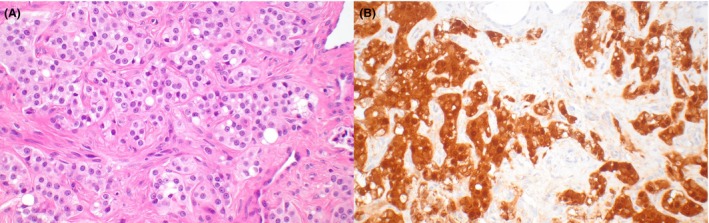
Sertoli cell tumour not otherwise specified (NOS). (**A**) Sertoli cell tumour NOS shows evidence of sex cord differentiation, with tubules, trabeculae, nests and/or cords of epithelioid cells with a moderate amount of eosinophilic cytoplasm that often contains vacuoles. (**B**) More than 90% of primary (i.e., non‐metastatic) Sertoli cell tumours NOS are driven by gain‐of‐function *CTNNB1* alterations or, rarely, by loss‐of‐function *APC* variants; these variants correlate with diffuse nuclear expression of beta‐catenin. In contrast, Leydig cell tumours with *CTNNB1* variants typically show focal or multifocal (rather than diffuse) nuclear expression of beta‐catenin (see Figure 3B).

Sertoli cell tumours NOS show evidence of sex cord differentiation, which is reflected by the presence of tubular, corded or trabecular growth patterns (Figure 5A).^45^ They are mostly solid but may sometimes show different degrees of cystic change, with occasional neoplasms containing cystic spaces that can be identified macroscopically.^46^ The cells of Sertoli cell tumour NOS exhibit a lesser amount of cytoplasm than those of LCTs, and the presence of lipofuscin pigment is uncommon. Signet ring‐like cells with a single intracytoplasmic vacuole devoid of mucin are very common in Sertoli cell tumor NOS. Although TSCSTs with pure signet ring cell‐like morphology are currently regarded as a distinct entity in the latest World Health Organisation classification (2022),^47^ we interpret them as a pattern of Sertoli cell tumour NOS (please see molecular characteristics below). Unlike typical examples of LCT, which show a minimal amount of stroma, Sertoli cell tumours typically exhibit a moderate to abundant amount of stroma with variable degrees of hyalinisation. In some rare cases, the stroma is extensively hyalinised and represents most of the tumour volume; these neoplasms have been described as ‘sclerosing’ Sertoli cell tumour in the literature.^48^, ^49^, ^50^ Parameters such as patient age, tumour size (different cut‐offs of 2.4 and 5 cm have been proposed), mitotic activity (>5 mitoses per 10 high power fields), lymphovascular invasion, necrosis and nuclear atypia seem to correlate with aggressive behaviour, but clinically validated parameters to assess the risk of malignancy are lacking.^46^, ^51^, ^52^ Of particular significance is a subset of clinically malignant TSCSTs that show morphological overlap with seminoma and were originally interpreted as malignant Sertoli cell tumours NOS.^52^ These neoplasms are characterised by a nested architecture, the presence of mixed inflammatory infiltrates, thick collagenous stroma and a distinct immunoprofile; therefore, they likely represent a separate entity (inflammatory and nested testicular sex cord tumour; see below). Sertoli cell tumours NOS are typically positive for SF1 and, consistent with their sex cord phenotype, often express markers such as SOX9 and FOXL2.^14^

Sertoli cell tumour NOS is largely driven by gain‐of‐function *CTNNB1* mutations, which are identified in ~70% of these neoplasms overall.^53^, ^54^ These are clonal events that correlate with diffuse nuclear expression of beta‐catenin (Figure 5B),^53^ a relevant difference with *CTNNB1‐*altered LCTs.^25^ When the diagnosis is restricted to neoplasms with typical features, the vast majority of primary Sertoli cell tumour NOS (>90%) show evidence of *Wnt* pathway activation, mostly, albeit not exclusively, by gain‐of‐function *CTNNB1* exon 3 variants.^53^, ^54^ By contrast, after tumours with *EWSR1* rearrangements are excluded (see below), only ~50% of clinically malignant Sertoli cell tumours NOS show evidence of *Wnt* pathway activation by gain‐of‐function *CTNNB1* mutations. In these tumours, *CTNNB1* variants are typically present with multiple concurrent chromosomal aneuploidies and regional or focal amplification events involving cancer‐relevant genes such as *MDM2* and *TERT*. This suggests that a subset of clinically malignant Sertoli cell tumours NOS may result from progression of primary neoplasms with *Wnt* activation.^55^ There have been a few documented cases with diffuse nuclear beta‐catenin expression secondary to functional inactivation of *APC* in the context of a likely or confirmed germline *APC* variant [familial adenomatous polyposis (FAP)].^56^ This suggests that germline *APC* alterations may predispose patients to develop TSCSTs, although the association needs to be corroborated in future studies. Of note, tumours reported in two patients with known FAP were either bilateral or multifocal, suggesting that the presence of multiple synchronous Sertoli cell tumours NOS may indicate a possible germline *APC* alteration.^56^, ^57^ A study of a series of TSCSTs with pure signet ring cell‐like morphology has demonstrated that they also consistently harbour gain‐of‐function *CTNNB1* alterations, a finding that further supports their interpretation as a pattern of Sertoli cell tumour, NOS.^58^, ^59^

## Inflammatory and Nested Testicular Sex Cord Tumour

**Figure 6:**
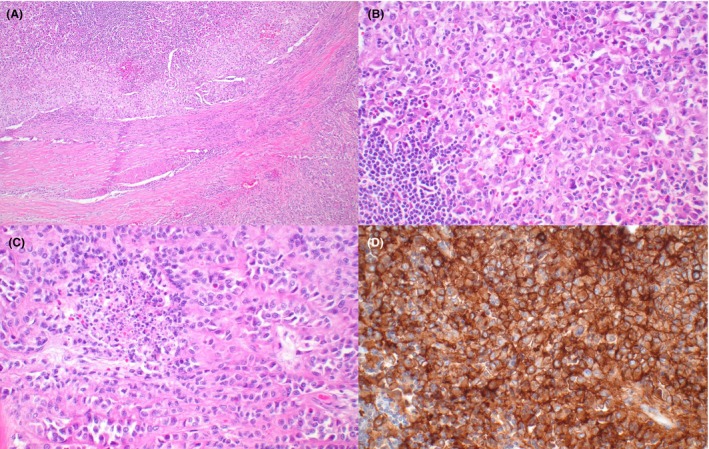
Inflammatory and nested testicular sex cord tumour. (**A**) These neoplasms are characterised by small to intermediate‐sized nests of tumour cells embedded in a thick collagenous stroma associated with prominent inflammatory infiltrates. (**B**) Tumour cells are intermediate‐sized, with granular eosinophilic or vacuolated cytoplasm and slightly irregular nuclei that contain vesicular chromatin and inconspicuous nucleoli. In addition to lymphocytes and plasma cells, the inflammatory infiltrates seen in these tumours typically include eosinophils (seen in the picture) and neutrophils. (**C**) These tumours typically show a low mitotic rate (<5 mitoses per 10 high‐power fields), but tumour necrosis is frequently found. (**D**) In addition to sex cord stromal tumour markers (SF1, inhibin), they frequently express CD30 (~80%, shown in the picture) and EMA (not shown). Inflammatory and nested testicular sex cord tumour appears to be invariably aggressive and shows a strong association with *EWSR1::ATF1* or *EWSR1* rearrangements

A subset of clinically malignant TSCSTs with morphologic resemblance to seminoma merits a separate discussion. These neoplasms, which were initially interpreted as examples of malignant Sertoli cell tumour NOS,^52^ are characterised by a solid nested growth pattern and the presence of variably prominent mixed inflammatory infiltrates containing lymphocytes, plasma cells, eosinophils and neutrophils (Figure 6 A‐C). Tumour cells exhibit a moderate amount of light eosinophilic to clear vacuolated cytoplasm and slightly irregular nuclei with small nucleoli. The nuclei of inflammatory and nested testicular sex cord tumour lack the angled (‘squared‐off’) outline that characterises the nuclei of seminoma. Although their mitotic activity is relatively low (most show less than 5 mitoses per 10 high‐power fields), they often exhibit other aggressive histopathologic features such as invasive growth beyond the testis and necrosis.^60^ These tumours are frequently misdiagnosed as seminoma due to their solid architecture and the presence of prominent inflammatory infiltrates.^52^ Interestingly, besides sex cord stromal tumour markers such as inhibin and SF1, they express CD30 (which has resulted in rare cases being misdiagnosed as embryonal carcinoma) and EMA (Figure 6D).^60^ Molecular analyses have revealed that most inflammatory and nested testicular sex cord tumors harbour *EWSR1* rearrangements and/or *EWSR1::ATF1* gene fusions.^60^, ^61^, ^62^, ^63^ Given their unique clinicopathologic and molecular features, it has been proposed that these neoplasms represent a distinct entity.^60^ The degree to which these testicular tumours overlap with recently described *EWSR1::ATF1*‐driven soft tissue tumours with epithelioid and round cell morphology is currently unknow and merits further investigation.^64^

## Large Cell Calcifying Sertoli Cell Tumour

Large cell calcifying Sertoli cell tumour (LCCSCT) is a specific subtype of Sertoli cell tumour with characteristic morphologic features that affects patients of a wide age range, from prepubertal children to adult men. LCCSCTs have been associated with the Carney complex at variable frequencies (from ~10% to ~40%),^65^, ^66^, ^67^ and rare cases have been reported in association with Peutz‐Jeghers syndrome and neurofibromatosis type 1.^68^, ^69^ However, those reported in the context of Peutz‐Jeghers syndrome are most likely driven by *STK11* alterations and may fall within the spectrum of tumours currently designated as intratubular large cell hyalinising Sertoli cell neoplasia (see corresponding section below).^70^ LCCSCTs associated with the Carney complex tend to affect children and adolescents, showing multifocal or bilateral testicular involvement and typically an indolent clinical behaviour.^71^ Patients may present with a testicular mass or manifestations of abnormal steroid sex hormone production, including gynecomastia and precocious isosexual pseudopuberty in children. Approximately 10% of LCCSCTs are clinically malignant; these tumours typically affect adult men (>25 years) and are largely sporadic.^72^, ^73^

**Figure 7:**
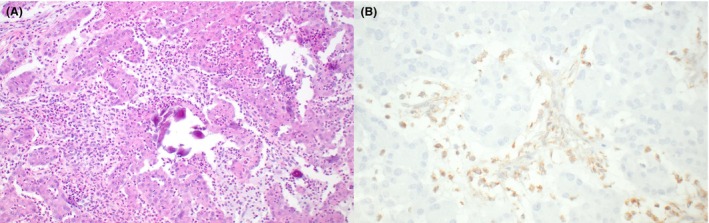
Large cell calcifying Sertoli cell tumour. (**A**) Large cell calcifying Sertoli cell tumour is characterised by nests, trabeculae, cords and/or solid sheets of large polygonal cells with abundant bright eosinophilic cytoplasm and round regular nuclei with visible nucleoli. They often show a myxoid stroma that contains variably abundant neutrophils. Lymphocytic infiltrates are also commonly seen, especially at the periphery of the tumours (not shown). (**B**) Both sporadic and Carney complex–associated large cell calcifying Sertoli cell tumours are almost invariably associated with loss‐of‐function *PRKAR1A* variants, which correlate with loss of PRKAR1A expression (note the presence of retained expression in the neutrophils that infiltrate the stroma)

LCCSCTs are composed of solid sheets, nests, trabeculae and tubule‐like structures containing large polygonal cells with abundant bright eosinophilic cytoplasm. Nuclei are round and regular, with a single nucleolus that is easily identifiable at intermediate magnification (~20x). They often exhibit a moderate to abundant amount of stroma that is characteristically myxoid, containing neutrophils and scattered mulberry‐like laminated calcifications (Figure 7A). However, calcifications can be sparse or even absent in approximately 20% of LCCTs.^66^ Lymphocytic infiltrates are also common and tend to cluster at the periphery of the tumour. Intratubular components are sometimes identified, and at least one purely intratubular LCCSCT has been documented in the literature.^66^ Histopathologic findings associated with malignant clinical behaviour, first described by Kratzer *et al*., include: size >4 cm, extension beyond the testis, necrosis, nuclear atypia, vascular invasion and >3 mitoses per 10 high‐power fields.^72^, ^74^ Given that a recent study has documented metastasizing LCCSCTs lacking aggressive histopathologic features in adult patients, it has been suggested that all neoplasms presenting in men older than 25 years should be considered potentially aggressive and warrant follow‐up.^73^

Studies performed in recent years suggest that the vast majority of LCCSCTs (>90%), including syndromic and sporadic cases, are associated with *PRKAR1A* alterations that result in functional inactivation of the gene.^66^, ^67^, ^75^, ^76^, ^77^ *PRKAR1A* variants are commonly the sole pathogenic genomic finding in clinically benign LCCSCTs without aggressive histopathologic features, whereas clinically malignant tumours tend to harbour additional alterations, including mutations, chromosomal aneuploidies and homozygous deletion of tumour suppressors (e.g., *CDKN1B/2A/2B*), among others.^67^, ^77^ Given the strong association between LCCSCT and *PRKAR1A* alterations, PRKAR1A immunohistochemistry represents an excellent diagnostic tool, with loss of expression of this marker being 93% sensitive for LCCSCT (Figure 7B). The specificity of PRKAR1A immunohistochemistry for distinguishing between LCCSCT and TSCSTs with overlapping morphology (such as LCTs and Sertoli cell tumour NOS) is also excellent (97%).^66^

## Intratubular Large Cell Hyalinising Sertoli Cell Neoplasia

**Figure 8:**
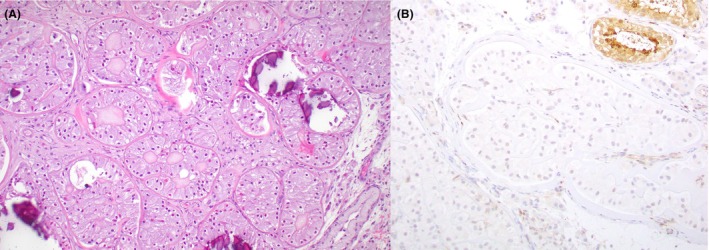
Intratubular large cell hyalinising Sertoli cell neoplasia. (**A**) These lesions are associated with the Peutz‐Jeghers syndrome and are typically bilateral and multifocal. They are largely microscopic (i.e., not mass‐forming), being composed of large epithelioid Sertoli cells with abundant bright eosinophilic cytoplasm and round regular nuclei typically (albeit not exclusively) confined to profiles of seminiferous tubules. They are associated with extracellular globular aggregates of eosinophilic basement membrane material that may calcify. (**B**) Given their association with the Peutz‐Jeghers syndrome, they are expected to be driven by loss‐of‐function *STK11* variants. Accordingly, a subset of ~60% demonstrates loss of expression of LKB1 protein (encoded by *STK11*). (**B**) *Courtesy of Dr. William J. Anderson, Brigham & Women's Hospital (Boston, MA)*

Intratubular large cell hyalinising Sertoli cell neoplasia is an entity that has been consistently associated with Peutz‐Jeghers syndrome in prior reports.^78^, ^79^, ^80^, ^81^ It affects children and young teenagers, who typically present with gynecomastia and bilateral testicular enlargement, but no distinct testicular mass.^82^, ^83^ Patients also show evidence of advanced bone age in the context of elevated serum estradiol levels.^84^ They are often diagnosed in testicular biopsies/partial orchiectomies as multifocal microscopic lesions characterised by large polygonal Sertoli cells with an abundant amount of bright eosinophilic cytoplasm that may contain vacuoles. The neoplastic cells are confined to profiles of seminiferous tubules (Figure 8A). Extracellular globular eosinophilic deposits of basement membrane material are common; these deposits often calcify, creating overlap with LCCSCT.^78^ Based on its association with Peutz‐Jeghers syndrome, intratubular large cell hyalinising Sertoli cell neoplasia is expected to harbour *STK11* alterations, but genetic analysis has been limited by the small size of these lesions. A recent study with immunohistochemistry has demonstrated that, unlike LCCSCT, a significant subset of intratubular large cell hyalinising Sertoli cell neoplasia (~60%) exhibits loss of expression of LKB1 (coded by *STK11*, Figure 8B).^85^ Of note, this study also showed that Peutz‐Jeghers‐associated TSCSTs with loss of STK11/LKB1 expression may include invasive components. This may explain prior reports of LCCSCTs in patients with Peutz‐Jeghers syndrome, given that the name ‘intratubular large cell hyalinising Sertoli cell neoplasia’ can only accommodate non‐invasive tumours. Hence, we believe that the nomenclature of Peutz‐Jeghers‐associated TSCSTs could be modified to include invasive tumours with *STK11* alterations (e.g., ‘large cell hyalinising Sertoli cell neoplasia/tumour’).

## Adult Granulosa Cell Tumour

Adult granulosa cell tumour of the testis represents, in the authors' opinion, a loosely defined, heterogeneous diagnostic category. It includes tumours that bear close resemblance to ovarian counterparts, as well as others with varied morphology that cannot be classified into one of the other histologic subtypes described in this review. Therefore, in our experience, many tumours that would otherwise remain unclassified are sometimes diagnosed as adult granulosa cell tumour based on the presence of some subtle non‐specific histologic findings, such as nuclear grooves. These tumours commonly present as intrascrotal masses,^86^, ^87^ but some exhibit manifestations of abnormal sex hormone production.^88^ A subset of adult granulosa cell tumours (~10% overall) show malignant clinical behaviour and metastasize.^88^, ^89^, ^90^

The lenient use of this diagnostic category has resulted in a markedly wide morphologic spectrum, including solid and cystic tumours with varied architecture and cytomorphology. Growth patterns include solid sheets, nests, fascicles, trabeculae, follicles and microfollicles (Call‐Exner bodies), among others.^91^ Tumours are composed of round, ovoid, and/or spindle neoplastic cells with a small amount of eosinophilic cytoplasm and variably irregular nuclei containing open chromatin and small nucleoli. The most commonly recurrent feature in these tumours is the presence of intranuclear grooves.^92^ Pathologic features associated with aggressive behaviour have been described in a limited number of cases, but the available data are limited.^88^, ^91^, ^92^, ^93^, ^94^

Well over 90% of ovarian adult granulosa cell tumours harbour a recurrent gain‐of‐function *FOXL2* mutation (p.C134W), and tumours that lack this alteration can often be re‐classified upon re‐review of their histopathologic features.^95^, ^96^, ^97^, ^98^ Testicular adult granulosa cell tumours are comparatively heterogeneous, and the hotspot *FOXL2* mutation mentioned above appears to be quite infrequent, having been documented only in anecdotal cases.^99^, ^100^ The only recurrent finding in a series of testicular adult granulosa cell tumours was loss of 22q, most likely a random event given the high frequency of this copy number alteration in cancer databases.^101^

## Juvenile Granulosa Cell Tumour

Juvenile granulosa cell tumours of the testis bear a great morphologic resemblance to ovarian counterparts, but they also show important clinical and molecular differences. For instance, unlike ovarian juvenile granulosa cell tumours, those arising in the testis represent an infantile disease that appears to be invariably benign.^88^, ^102^, ^103^ These tumours present largely during the first year of life (90% in infants ≤ 6 months), mostly as testicular masses,^83^, ^103^, ^104^, ^105^, ^106^, ^107^ with gynecomastia being identified in rare patients.^102^

**Figure 9:**
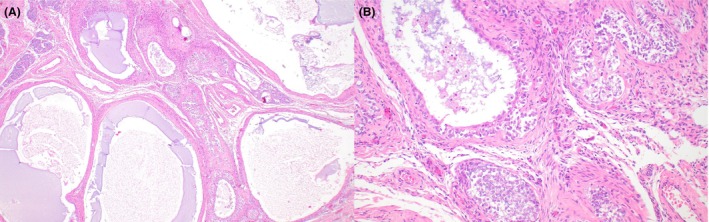
Juvenile granulosa cell tumour. (**A**) These benign infantile testicular tumours are typically multicystic or solid and cystic. They are composed of variably prominent follicles containing eosinophilic or basophilic secretions, separated by bands of fibrous stroma. (**B**) The cells lining the follicles are round and small, with scant eosinophilic cytoplasm and bland nuclei containing open chromatin. These tumours often exhibit solid nests of cells with intercellular accumulation of secretions similar to those seen in the lumina of the follicles (inferior part of the picture). Some neoplasms may also include solid areas with spindle‐cell morphology (not shown)

Macroscopically, juvenile granulosa cell tumours are well‐circumscribed, multicystic or solid and cystic. Individual cysts contain thin mucinous secretions and are separated by variably prominent bands of fibrous and/or myxoid stroma.^102^ Microscopically, tumours are composed of follicles containing eosinophilic or basophilic secretions, lined by one or more layers of small round to oval cells with regular nuclei and a small amount of eosinophilic cytoplasm (Figure 9). Areas of solid growth containing similar cells are frequently present; the solid components show intercellular spaces with accumulation of secretions, suggesting that follicles form by the coalescence of these small intercellular fluid‐filled spaces.^108^ Solid areas of juvenile granulosa cell tumours may contain bland neoplastic spindle cells. Some neoplasms show areas with microcystic (reticular) architecture that mimic yolk sac tumour.^102^ Of note, some neoplasms may show prominent mitotic activity (>5–10 mitoses per 10 high‐power fields), but this is not associated with aggressive clinical behaviour.^12^, ^102^ Necrosis, lymphovascular invasion, and involvement of the rete testis have been described in rare cases.^102^

Juvenile granulosa cell tumours of the ovary show recurrent findings including internal tandem duplications within the pleckstrin‐homology domain of *AKT1* and hotspot (codon 201) *GNAS* mutations.^109^, ^110^, ^111^ By contrast, these variants have not been identified in testicular counterparts, which show monosomy 10 as a recurrent finding instead (~60%).^112^

Given the important clinicopathologic and molecular differences between ovarian and testicular juvenile granulosa cell tumours (in particular, the invariably indolent nature of the later^112^), it has been recommended that this entity be re‐classified as ‘benign juvenile granulosa cell tumour of the testis’ (Table 2).^32^ The rationale for this recommendation is that the current nomenclature may lead clinicians to assume that these neoplasms are equivalent to ovarian juvenile granulosa cell tumours, resulting in over‐treatment and unnecessary workups.

**Table 2:** Recommendations for classification of testicular sex cord–stromal tumours from the testicular sex cord–stromal tumour (TESST) group (adapted)

Recommendation	Justification
Leydig cell tumours with FH deficiency should be classified as ‘FH‐deficient Leydig cell tumour’	To highlight tumours with potentially aggressive behaviour and an alteration that may prompt further genetic assessment
2 In TSCSTs without nuclear beta‐catenin expression, the diagnosis of Sertoli cell tumour NOS should be restricted to neoplasms with ‘typical’ morphology (e.g., tubular architecture)	Nuclear beta‐catenin expression in >90% of primary Sertoli cell tumours
3 Primary TSCSTs with unusual growth patterns and diffuse nuclear beta‐catenin expression can be favoured to represent Sertoli cell tumour NOS	Diffuse nuclear beta‐catenin is typical of Sertoli cell tumour NOS; methylation studies of a limited number of cases support this conclusion
4 Aggressive undifferentiated TSCSTs with diffuse nuclear beta‐catenin expression can be favoured to represent ‘undifferentiated Sertoli cell tumour NOS’	Diffuse nuclear beta‐catenin is typical of Sertoli cell tumour NOS; ~50% of malignant Sertoli cell tumours NOS are driven by *CTNNB1* alterations
5 Sclerosing Sertoli cell tumour and signet ring stromal tumour should be considered patterns of Sertoli cell tumour NOS	Similar clinicopathologic and molecular features
6 TSCSTs with *EWSR1::ATF1* or *EWSR1* rearrangement represent a distinct entity (inflammatory and nested testicular sex cord tumour)	Invariably aggressive, unique clinicopathologic features
7 Testicular stromal neoplasms with pure spindle morphology can be classified as ‘spindle cell gonadal stromal tumour’	Fibroma/thecoma and myoid gonadal stromal tumour demonstrate significant clinicopathologic overlap and can be difficult to distinguish
8 Juvenile‐type granulosa cell tumour of the testis should be re‐classified as ‘benign juvenile‐type granulosa cell tumour’	To avoid confusion with ovarian homonyms, which show malignant potential (~10%)
9 Diagnosis of testicular adult‐type granulosa cell tumour should be restricted to tumours morphologically equivalent to ovarian counterparts. *FOXL2* p.C134W may also aid in classifying these tumours	Used in the past as diagnosis of exclusion, creating morphologic and molecular heterogeneity
10 ‘Mixed sex cord–stromal tumours’ includes tumours with sex cord and stromal neoplastic components not classifiable into other specific subtypes	Diagnosis of exclusion. Although the initial recommendations suggested that missed tumours with nuclear beta‐catenin expression be considered a subtype of Sertoli cell tumour NOS, it is also reasonable to consider them mixed sex cord–stromal tumours
11 Testicular Sertoli‐Leydig cell tumour is a distinct but exceedingly rare entity	Exceedingly rare cases with analogous clinicopathologic and/or molecular features to ovarian counterparts have been documented in the literature

## Gonadal Stromal Tumours with Pure Spindle Cell Morphology: Myoid Gonadal Stromal Tumour and Fibroma/Thecoma

This category encompasses pure spindle cell neoplasms that are classified as myoid gonadal stromal tumour (MGST) and fibroma/thecoma in the latest World Health Organization (2022) blue book.^1^, ^47^ These pure spindle cell stromal tumours appear to be invariably benign and, in the authors' opinion, their distinction can be subjective in many instances. In fact, a recent study demonstrated that these diagnoses are not reproducible even among expert uropathologists.^113^ MGST is thought to derive from primitive peritubular myoid cells and, by definition, co‐expresses smooth muscle actin and S100.^114^, ^115^, ^116^, ^117^ Fibromas are pure spindle cell tumours that can occur entirely within the testicular parenchyma or be attached to the tunica albuginea. It has been proposed that those that are entirely intraparenchymal represent the testicular counterparts of ovarian fibromas, while those associated with the albuginea should be considered fibromas of the testicular tunics.^118^ These gonadal stromal tumours affect postpubertal patients, from teenagers to elderly adults, and typically present as a testicular mass.^119^, ^120^, ^121^

**Figure 10:**
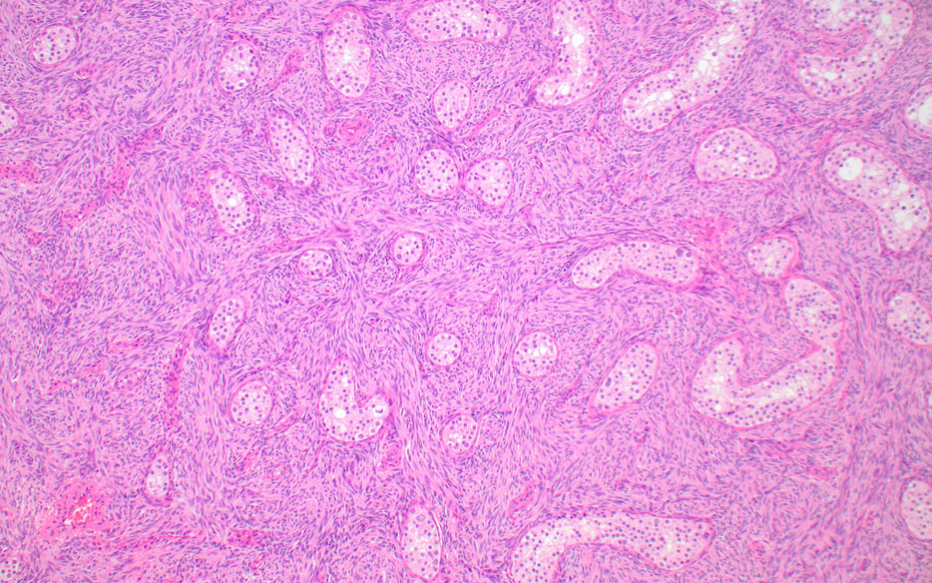
Spindle cell gonadal stromal tumours. This category comprises a spectrum of pure spindle cell lesions that have been classified as fibroma/thecoma and myoid gonadal stromal tumour in the literature. They are composed of bland spindle cells organised in short fascicles and/or whorls within a variably prominent collagenous stroma. These benign tumours tend to grow entrapping normal structures such as seminiferous tubules and *rete testis*; however, destructive invasive growth is not seen

Macroscopically, they are well‐circumscribed solid nodules with a firm cut surface that can show a whorled appearance.^119^ Microscopically, they are pure spindle cell neoplasms composed of bland spindle cells with elongated nuclei and small nucleoli. Most cases show a relatively low proliferative rate (≤5 mitoses per 10 high power fields), although occasional tumours may exhibit brisker mitotic activity, a finding that does not impact clinical outcome.^119^ In addition, genitourinary pathology experts have occasionally encountered fibromas/thecomas with foci of necrosis. Although destructive/invasive growth is typically not a feature of these neoplasms, extratesticular extension has been occasionally reported.^119^, ^122^ Lymphovascular invasion has not been domcumented in fibromas and MGSTs, to the best of our knowledge.^115^, ^114^, ^117^, ^118^, ^119^ They tend to grow entrapping normal (i.e., non‐neoplastic) structures, such as *rete testis* and seminiferous tubules, without destroying them (Figure 10).^117^, ^119^, ^120^

In this review, we have included MGST and fibroma/thecoma under a single section because they demonstrate significant clinicopathologic and molecular overlap. However, prototypical cases of these entities do show differences that are worth highlighting. Fibromas/thecomas often include areas with storiform or herringbone architecture and may contain cells with epithelioid morphology, which are not typical of MGST. Fibromas/thecomas also exhibit a somewhat prominent collagenous stroma with collagen plaques, whereas most MGSTs show an inconspicuous stromal component.^120^ These tumours often express sex cord stromal tumour markers such as inhibin, calretinin and SF1. A potentially useful marker is SOX9, which has been consistently negative in a small number of MGSTs (0 of 3) and positive in most fibromas (3 of 4) tested in a prior study.^115^ As mentioned above, by definition, MGSTs co‐express S100 and smooth muscle actin; although these are relatively non‐specific markers, fibromas are not expected to express S100.^114^, ^119^ Reticulin histochemical stain may be useful to distinguish fibroma/thecoma (in which reticulin fibres surround individual cells) from adult granulosa cell tumour with spindle cell morphology (in which reticulin fibres surround nests of cells).^123^, ^124^ Of note, the authors have encountered intraparenchymal pure spindle cell tumours that express sex cord‐stromal tumour markers such as SF1 but cannot be definitely classified as fibroma/thecoma or MGST. In this scenario, we use the terms ‘spindle cell gonadal stromal tumour’ or ‘spindle cell gonadal stromal tumour, not otherwise specified’.

A limited number of genomic studies have not identified recurrent mutations in MGSTs.^117^, ^, 125^ Instead, they are characterised by multiple chromosomal gains suggestive of a shift in ploidy.^125^, ^126^ Interestingly, similar findings have been reported in testicular tumours with pure or predominant spindle cell components that were originally classified as adult granulosa cell tumour, unclassified sex cord stromal tumour and Sertoli‐stromal cell tumour.^101^, ^126^, ^127^, ^128^ This further highlights the overlap that exists between testicular neoplasms with spindle cell morphology and suggests that, at least for now, there is limited evidence to support that they represent clearly distinct entities. Therefore, it has been suggested that a diagnosis of ‘spindle cell gonadal stromal tumour’ is adequate for lesions that fall within the spectrum of MGST and fibroma/thecoma, when a distinction between these entities cannot be confidently made. The most important message for treating clinicians is that, based on what we know so far, these neoplasms appear to be invariably benign and are therefore cured by complete surgical resection.

## Mixed Sex Cord Stromal Tumour and Sex Cord Stromal Tumour NOS

Mixed TSCST and TSCST NOS represent largely diagnoses of exclusion that require ruling out the histologic subtypes described above. Mixed TSCST are characterised by a combination of sex cord and stromal elements, a definition that encompasses tumours with Sertoli/sex cord and Leydig cell components as well as others with Sertoli/sex cord and spindle cell stromal components.^129^, ^130^, ^131^ Most mixed TSCSTs are composed of spindled stromal cells and a sex cord component that typically consists of cords, trabeculae, ribbons or, rarely, tubules of immature Sertoli cells. The spindle cells are typically bland and resemble those of MGST or fibroma/thecoma. The spectrum of neoplasms with sex cord and spindle cell components includes tumours that have been described as ‘Sertoli‐stromal cell tumour’ in the literature.^128^ As for neoplasms composed of Sertoli and Leydig cells, it is thought that at least a subset may represent Sertoli cell tumours NOS with entrapped non‐neoplastic Leydig cells, although rare *bona‐fide* counterparts to ovarian Sertoli‐Leydig cell tumours have been documented.^128^ TSCST NOS represents a heterogeneous group that comprises neoplasms with a wide range of morphologies, which cannot be classified as one of the specific histologic subtypes mentioned above.^132^ This category includes tumours with pure epithelioid and pure spindle cell morphology that vary from indolent to highly aggressive.^127^, ^133^, ^134^, ^135^

**Figure 11:**
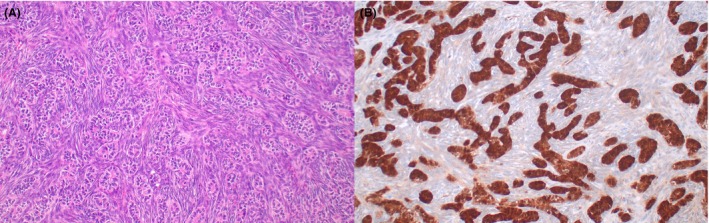
Mixed sex cord stromal tumours. (**A**) Most tumours within this category are characterised by a mixture of sex cord elements composed of immature Sertoli cells intermingled with a stromal component represented by bland spindle cells. (**B**) *CTNNB1* alterations have been documented in a small subset of these tumours, which demonstrate nuclear beta‐catenin expression restricted to the sex cord component

Mixed TSCSTs and TSCSTs NOS have shown heterogeneous genomic findings, in line with their varied phenotypes. A subset harbours gain‐of‐function *CTNNB1* alterations, and one study has suggested that some of them may represent examples of Sertoli cell tumour NOS with unusual morphology.^127^ Among mixed TSCSTs (Sertoli‐stromal cell tumour), those with *CTNNB1* mutations tend to show nuclear beta‐catenin expression restricted to the sex cord components (Figure 11A,B).^14^, ^54^, ^128^, ^136^ Tumours with pure or predominant spindle cell components tend to show multiple chromosomal gains like those described in MGST.^125^, ^127^ Of note, one Sertoli‐Leydig cell tumour of the testis bearing close morphologic resemblance to ovarian Sertoli‐Leydig cell tumour was reported to harbour a hotspot (RNAse IIIb domain) *DICER1* variant.^128^

## Potential Therapeutic Implications of New Molecular Findings

Given that SCSTs are notoriously resistant to standard chemotherapy, the new molecular findings offer potential targets for novel therapeutic agents. Specifically, a few actionable molecular findings have been identified, including *CDK4* (target for cell cycle inhibitors), *RICTOR*, *NF2* and *PTEN* (targets for mTOR inhibitors), *PTCH1* (target for hedgehog pathway inhibitors) and *BAP1* [target for poly(ADP‐ribose) polymerase (PARP) inhibitors].^27^ Recently, a patient with a malignant Leydig cell tumour and response to a *CDK4* inhibitor has been documented.^137^ The high frequency of beta‐catenin alterations and Wnt pathway activation in Sertoli cell tumours NOS suggests that they could respond to Wnt pathway inhibitors that are currently being assessed in clinical trials (mostly for colorectal cancer).^138^ Anti‐VEGF drugs such as lenvatinib or bevacizumab combined with immunotherapy or EGFR inhibitors have shown encouraging results in FH‐deficient renal cell carcinoma, suggesting that they may also derive some benefit for patients with FH‐deficient Leydig cell tumours.^139^ Partial responses to immune checkpoint inhibitors have been anecdotally reported in patients with malignant SCSTs.^140^ Inflammatory and nested testicular sex cord tumours typically express CD30 diffusely, raising the possibility that brentuximab vedotin, an anti‐CD30 antibody–drug conjugate, may play a role in the treatment of these patients in combination with surgery.^141^ Trials with bevacizumab are now available for patients with ovarian adult granulosa cell tumours, although it is uncertain if patients with testicular counterparts are potential candidates, considering the differences between adult granulosa cell tumours of the ovary and testis.^142^ It is important to highlight that these therapies should be considered experimental for TSCSTs, as there is currently minimal to null clinical data to support their use.

## Conclusions

TSCSTs are currently classified based on morphology, with some histologic subtypes being defined by their similarity to ovarian homonyms.^45^ Although immunohistochemistry is often useful to determine sex cord and/or stromal lineage,^143^ most SCSTs lack specific immunomarkers (Table 3). For instance, the immunoprofiles of most testicular SCSTs show substantial overlap, with purported ‘sex cord markers’ such as WT1, FOXL2 and SOX9 being expressed in tumours with seemingly pure stromal phenotype and vice‐versa. A few tumour types with relatively specific markers represent relevant exceptions, such as large cell calcifying Sertoli cell tumour (characterised by loss of PRKAR1A expression), intratubular large cell hyalinising Sertoli cell neoplasia (characterised by loss of STK11 expression) and inflammatory and nested testicular sex cord tumour (characterised by co‐expression of EMA and CD30). However, these antibodies are not widely available and the sensitivity of some of them, such as CD30 and STK11, is below 90%.^60^, ^85^

**Table 3:** Immunohistochemistry features of the most common testicular sex cord–stromal tumours

Tumour	Beta‐catenin	SF‐1	Inhibin	Calretinin	WT1	SOX9	FOXL2	Other
Leydig cell tumour	Membrane or may be focal/multifocal and heterogeneous in <50% of the tumour (nuclear)	+/‐	+/‐	+/‐	‐/+	‐/+	‐/+	
Sertoli cell tumour, not otherwise specified	+ diffuse (nuclear)	+/‐	+/‐	+/‐	+/‐	+/‐	+/‐	
Inflammatory and nested testicular sex cord stromal tumour	− (membrane)	+/‐	+/‐	‐/+	+/‐			EMA +, CD30 +
Large cell calcifying Sertoli cell tumour	− (membrane)	+/‐	+/‐	+/‐	‐/+	+/‐	‐/+	Loss of PRAKAR1A, S100 +
Large cell hyalinising Sertoli cell neoplasia	− (membrane)	+/‐	+/‐	+/‐				Loss of STK11
Adult granulosa cell tumour	− (membrane)	+/‐	+/‐	+/‐	+/‐	‐/+	+/‐ (but does not correlate^a^ with mutational status)	
Juvenile granulosa cell tumour	− (membrane)	+/‐	+/‐	+/‐		+/‐	+/‐	
*Spindle cell gonadal stromal tumours*: fibroma/thecoma	− (membrane)	+/‐	+/‐	‐/+	+/‐	+/‐	+/‐	
*Spindle cell gonadal stromal tumours*: MGST	− (membrane)	+/‐	+/‐	‐/+	‐/+	‐/+	+/‐	Co‐expression of S100 and SMA

MGST, myoid gonadal stromal tumour.

^a^ Not a mutation‐specific primary antibody.

**Figure 12:**
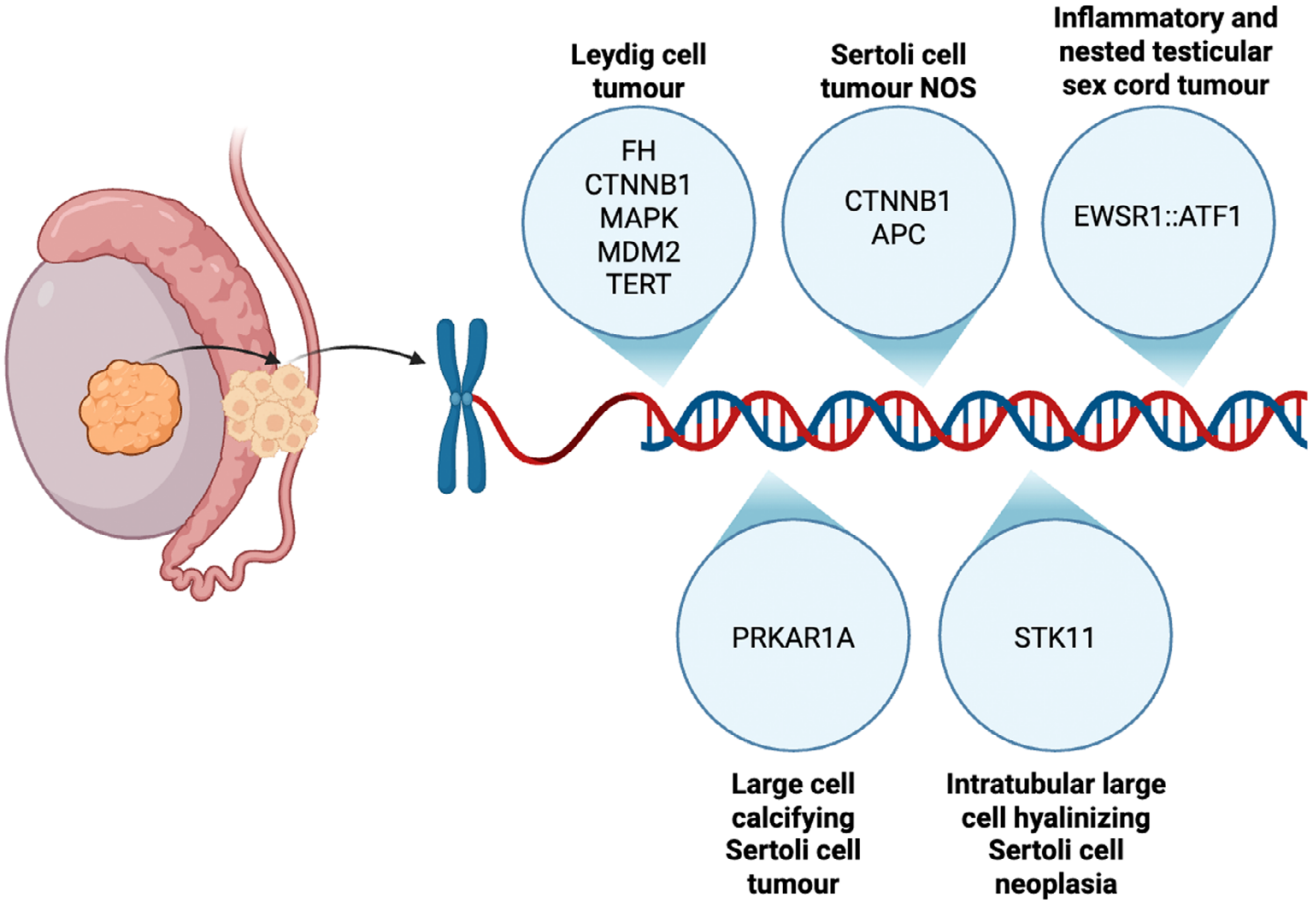
Summary of most recurrent molecular alterations in testicular sex cord–stromal tumours

Recently, molecular studies (Figure 12) have started to reveal the genomic complexity of these tumours, providing new data to refine their classification and, possibly, improve prognostication. Consequently, the two largest uropathology societies, the International Society of Urological Pathology (ISUP) and the Genitourinary Pathology Society (GUPS), have sponsored meetings of an international group of experts (Testicular Sex Cord Stromal Tumour: TESST) with the objective of issuing recommendations for classification and workup of TSCSTs.^32^ A summary of these recommendations is provided in Tables 2 and 4. Despite the recent advances, significant work needs to be done in the field to further understand the molecular alterations that underlie pathogenesis and progression, as well as to refine the nosology of these neoplasms. For instance, it is still uncertain how testicular adult granulosa cell tumours should be defined or how many different entities are included under the current categories 'mixed TSCST' and 'TSCST NOS'. Importantly, more work is needed to identify parameters that can accurately predict malignant potential and, therefore, improve patient management.

**Table 4:** Recommendations for workup and clinicopathologic assessment of testicular sex cord–stromal tumours from the testicular sex cord–stromal tumour (TESST) group

Recommendation	Justification
Immunohistochemistry should be performed on most TSCSTs	Exposure to these tumours is limited in most practices. General markers to determine sex cord–stromal origin is recommended (e.g., inhibin, SF1)
2 Subclassification of TSCSTs is important for clinical management	Predicting risk of malignancy and syndromic associations are different for different tumour types
3 Risk of recurrence/metastasis depends in part on the histologic subtype	TSCST types vary from invariably benign (e.g., juvenile granulosa cell tumour), to predominantly benign (e.g., Leydig cell tumour) and almost invariably aggressive (e.g., inflammatory and nested testicular sex cord tumour)
4 Non‐metastatic TSCSTs of postpubertal patients should be classified as ‘low risk’ or ‘high risk’ of malignant behaviour (instead of ‘malignant’ or ‘benign’)	Correlation between clinicopathologic findings and clinical behaviour is imperfect
5 The size threshold that is considered a ‘risk factor’ should be specific for each tumour type	Different size cut‐offs proposed for different entities
6 Age is a potential risk factor for malignant behaviour	Most malignant TSCSTs occur in adult patients
7 The pTNM system used to stage germ cell tumours should not be used for TSCSTs	Different biology (most TSCSTs are indolent). Some parameters are not easily extrapolated to TSCSTs
8 FH/MDM2 analysis is recommended in primary Leydig cell tumours with aggressive histopathologic findings	These alterations are enriched in aggressive Leydig cell tumours
9 FH immunohistochemistry is desirable in metastasizing Leydig cell tumours	To identify patients that may need further genetic assessment
10 TSCSTs with nested architecture and inflammatory infiltrates (mimicking seminoma) should be assessed for *EWSR1* rearrangements or *EWSR1::ATF1*	These alterations are highly recurrent in inflammatory and nested testicular sex cord tumour
11 Multifocal or bilateral Sertoli cell tumour NOS should undergo molecular assessment	Rare bilateral/multifocal Sertoli cell tumours NOS are associated with germline *APC* variants
12 Assessment of *PRKAR1A* status is desirable, albeit not necessary, to support a diagnosis of large cell calcifying Sertoli cell tumour (by IHC and/or molecular studies)	Loss of PRKAR1A function is seen in >90% of large cell calcifying Sertoli cell tumour

The information provided in this review is intended to bring the reader up to date with the new developments in the field and provide some practical guidance for the diagnosis of these rare tumours. However, we also hope that the readers can identify areas where knowledge is lacking, opening potential avenues for future research.

## Author contributions

Review of the literature and writing: JL and AMA. The authors read and approved the final manuscript.

## Funding information

None.

## Disclosures

In Figure 2B, the light curves were modified for better visualisation of the nuclei by the readers.

## Data Availability

Data sharing is not applicable to this article as no datasets were generated or analysed during the current study (review article).
